# Endocrine implications of bariatric surgery: a review on the intersection between incretins, bone, and sex hormones

**DOI:** 10.14814/phy2.14111

**Published:** 2019-05-27

**Authors:** Isabel Casimiro, Susan Sam, Matthew J. Brady

**Affiliations:** ^1^ Section of Endocrinology, Diabetes & Metabolism University of Chicago Chicago Illinois

**Keywords:** Bariatric surgery, GLP‐1, incretins, obesity

## Abstract

Bariatric surgery is now the most widely used intervention for the treatment of human obesity. A large body of literature has demonstrated its efficacy in sustained weight loss and improvement in its associated comorbidities. Here, we review the effect of bariatric surgery in gut hormone physiology, bone remodeling and the reproductive axis. Rapid improvements in insulin release and sensitivity appear to be weight loss independent and occur immediately after surgery. These effects on pancreatic beta cells are mostly due to increased gut hormone secretion due to augmented nutrient delivery to the small intestine. Bone remodeling is also affected by gut hormones. Phenotypic skeletal changes observed in mice deficient in GLP‐1 or GIP suggest that increased incretins may improve bone density. However, these positive effects may be counterbalanced by the association between weight loss and a reduction in bone density. Finally, studies have shown a marked improvement following bariatric surgery in infertility and PCOS in women and hypogonadism in men. Thus, the net effect on endocrine systems after bariatric surgery will likely vary on an individual basis and depend on factors such as comorbidities, peri‐menopausal state, amount of weight loss, and likelihood to adhere to vitamin supplementation after surgery.

## Introduction

Gastric bypass surgery leads to dramatic weight loss and improvement in insulin resistance to the point of the resolution of type 2 diabetes in a majority of cases (Sjostrom et al. 2014). These effects are due in large part to the anatomic changes that result in restrictions in food consumption, reduced appetite, and the change in gut hormone secretion that lead to profound overall systemic metabolic improvements (Holst et al. 2018). The types of bariatric surgeries that result in significant gut hormone changes include the Roux‐en‐Y gastric bypass, duodenal switch, biliopancreatic diversion, jejunoileal bypass, and the sleeve gastrectomy (Folli et al. 2012). Many other reviews have covered the effects of bariatric surgery on weight loss and improvements in systemic insulin sensitivity (Buchwald et al. 2004; Kohli et al. 2011; Dirksen et al. 2012; Nudel and Sanchez 2019; Sljivic and Gusenoff 2019). The focus of the current review will be to summarize how the physiological changes that have been shown to arise from sustained weight loss after bariatric surgery affect other interrelated endocrine processes such as bone remodeling and improve obesity‐related hypogonadal dysfunction.

The rise of obesity has reached epidemic proportions worldwide and is associated with significant comorbidities including type 2 diabetes mellitus (T2DM), hyperlipidemia, hypertension, obstructive sleep apnea, coronary heart disease, and insulin resistance. Bariatric surgery is an effective procedure for weight loss in obese patients that has become more widespread. Those who may be considered for weight loss surgery include obese patients with a body mass index (BMI) above 40, or those with a BMI between 35 and 39.9 and a severe obesity‐related comorbidity such as diabetes. Those with a BMI between 30 and 34.9 and poorly controlled diabetes can also be considered. BMI cutoffs may be lowered by 2.5 for patients of Asian descent (Rubino et al. 2016). The types of bariatric surgery procedures can be divided into restrictive, malabsorptive or combined procedures. The sleeve gastrectomy (SG) now a stand‐alone procedure that was initially performed as the first‐stage procedure for the duodenal switch (DS), is a restrictive surgery that limits the intake of solid food and calories by significantly reducing the size of the stomach by over 80% by creating a vertical tube from the stomach. Its prevalence has been rapidly increasing over the last two decades and is the most common procedure performed in the United States, Canada, and Asia (Angrisani et al. 2015). The Roux‐en‐Y‐gastric bypass (RYGB) is a combined malabsorptive and restrictive procedure that involves dividing the stomach into a small upper gastric pouch that is connected to the lower part of the bowel therefore bypassing a major portion of the stomach and the upper part of the intestine (Fobi 2004; Sljivic and Gusenoff 2019). Since the most common bariatric surgeries performed at this time are the SG and RYGB, this review will focus on these procedures. The malabsorptive procedures include the jejunoileal bypass which was the first operation used for the management of obesity and involves bypassing > 90% of small bowel leaving only about 40 cm of exposed small bowel for ingested food (Payne and Dewind 1969; Fobi 2004). The biliopancreatic diversion involves creating a small gastric pouch from the stomach that is connected to the final segment of the small intestine, bypassing the entire upper part of the small intestine. The biliopancreatic diversion with a duodenal switch involves preserving the stomach pylorus by a creating a sleeve gastrectomy and an intestinal bypass so that about two‐thirds or more of the intestine is bypassed (Fobi 2004).

## Gut Incretin Hormones

Incretins are endocrine hormones secreted by the enteroendocrine cells into the general circulation that stimulate pancreatic beta cells to secrete more insulin in response to a glucose load (Elrick et al. 1964). Glucagon‐like peptide‐1 (GLP‐1) and glucose‐dependent insulinotropic polypeptide (GIP), also known as gastric inhibitory polypeptide, were first identified after the 1970s (Rehfeld 2018). These incretins rise in the presence of nutrients in the intestinal lumen (Drucker and Nauck 2006). The L cell found in the ileum and large intestine secretes GLP‐1 and peptide YY (PYY) as well as cholecystokinin (CCK). CCK promotes gallbladder contraction, and along with PYY they slow gastric emptying and act as a satiety signal to improve glycaemia. The K cell, which resides in the proximal small intestine, secretes GIP in response to glucose and lipid stimulation and also secretes CCK (Tseng et al. 1994; Hutch and Sandoval 2017). Ghrelin, is secreted by the gastric and duodenal enteroendocrine cells and is the only orexigenic hormone produced in the GI tract that stimulates appetite and increased food intake. Upon secretion, GLP‐1 and GIP bind to G‐protein‐coupled receptors on beta cells that result in changes in cAMP and intracellular calcium, both of which increase insulin vesicular release. Shortly after stimulation, GLP‐1 and GIP are rapidly degraded by the ubiquitous dipeptidyl peptidase‐4 (DPP‐4) enzyme (Deacon 2005). GLP‐1 is a major regulator of satiety and glucose homeostasis. T2DM leads to the reduction in GLP‐1 levels and the meal‐stimulated response of GLP‐1 is also significantly reduced in these patients (Toft‐Nielsen et al. 2001). Incretin‐based therapies such as GLP‐1 agonists or DPP‐4 inhibitors have proven to be an effective tool for improving glycemic control (Davidson 2010; Htike et al. 2017). In addition, treatment with GLP‐1 receptor agonists, such as liraglutide, has been found to be effective for sustained weight loss (Khera et al. 2016).

Marked changes in gut hormone secretion occur after gastric bypass surgery and these hormonal changes are largely influenced by the type of bariatric surgery. These include a significant increase in GLP‐1 secretion just days after bariatric surgery in both RYGB and SG (Hutch and Sandoval 2017; Holst et al. 2018). The degree by which GLP‐1 rises after RYGB and SG is not seen in patients with a similar degree of weight loss achieved by caloric restriction (Laferrere et al. 2008). The increase in GLP‐1 and PYY secretion is thought to be due to the increased delivery of large nutrient loads to the distal gut containing a high number of GLP‐1 and PYY producing L cells as a result of intestinal rearrangement in RYGB and gastrectomy in SG (Gribble and Reimann 2019; Larraufie et al. 2019), with the stimulus being accelerated exposure and absorption of nutrients to the distal enteroendocrine cells (Jorgensen et al. 2012; Jacobsen et al. 2013; Kuhre et al. 2017; Smith et al. 2018). This phenomenon is known as the “hindgut hypothesis,” which states that improved glucose metabolism after bariatric surgery is due to the expedited delivery of nutrients resulting in an increased amount of distal ileum incretins like PYY and GLP‐1 (Patriti et al. 2007; Melissas et al. 2013; Pok and Lee 2014). Indeed, changes in the secretion of gut hormones after gastric bypass surgery are in part responsible for the mechanism of weight loss and resolution of T2DM after gastric bypass surgery (le Roux et al. 2006; Jorgensen et al. 2012; Holst et al. 2018). The early improvement in hepatic insulin resistance occurs within a few days of bariatric surgery with a reduction in HOMA‐IR of about 50% of preoperative values after one week in patients with T2DM who underwent RYGB (Jorgensen et al. 2012). Several studies that have investigated the mechanism accounting for improved glucose tolerance have implicated the acute rise in GLP‐1 in humans (Kashyap et al. 2010; Reed et al. 2011; Umeda et al. 2011; Jorgensen et al. 2012) and in rats (Duan et al. 2014). In mouse studies, wild‐type littermates and GLP‐1 receptor knockout mice both lost equal amounts of weight after RYGB and SG, suggesting GLP‐1 alone is not necessary for weight loss. However, multiple studies have shown that GLP‐1 signaling is a crucial player in the improved insulin sensitivity seen after bariatric surgery. GLP‐1 increases insulin secretion in a dose‐dependent manner and beta cell sensitivity to glucose is also increased by increased GLP‐1 levels after bariatric surgery (Kjems et al. 2003; Larraufie et al. 2019). Furthermore, GLP‐1 receptor (GLP‐1R) blockade impairs RYGB‐mediated improved glucose tolerance (Jorgensen et al. 2013) and blocks hyperinsulinemia after SG (Larraufie et al. 2019). GIP stabilizes glucose levels by enhancing glucose‐stimulated insulin secretion and promoting the secretion of glucagon when glucose is low. The role of GIP after gastric bypass surgery has been less clear, with studies showing inconsistent elevations in GIP levels or no changes after gastric bypass surgery (Falken et al. 2011; Jorgensen et al. 2012) The discrepancies in GIP levels may be due to the different surgical techniques used, since levels of GIP are correlated with the length of intestine that is exposed to ingested nutrients (Larraufie et al. 2019). CCK levels increase after both RYGB and SG, and while several studies have shown a reduction in ghrelin after RYGB and a greater reduction after SG (Nannipieri et al. 2013), this decrease has been inconsistent among all studies (Dimitriadis et al. 2017). Caloric restriction and ensuing weight loss will account for further improvement in insulin sensitivity due to improved skeletal muscle and pancreatic function after bariatric surgery (Campos et al. 2010). Diet‐induced weight loss also results in an alteration of metabolic hormones. However, only a small fraction of obese people are able to maintain diet‐induced weight loss over the long term. The hormonal changes that occur in diet‐induced weight loss differ from bariatric surgery‐induced weight loss and are listed in Table 1 and do not include a marked rise in GLP‐1. While caloric restriction also results in a reduction of leptin, the levels of ghrelin rise and PYY and CCK levels decrease, changes which likely contribute to regaining lost weight (Fruhbeck et al. 2004; Sumithran et al. 2011; Ramon et al. 2012; Nannipieri et al. 2013; Yang et al. 2014; Yousseif et al. 2014; Dimitriadis et al. 2017; Hutch and Sandoval 2017).

**Table 1:**
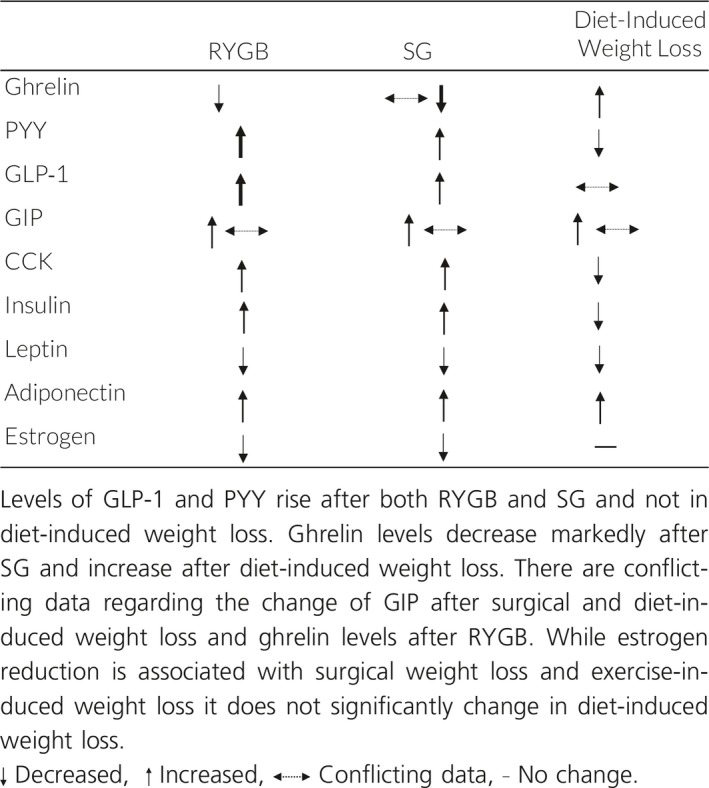
Effects of RYGB, SG and diet‐induced weight loss on incretins and hormones

### Relationship between incretins, adipokines, and bone remodeling

Bone remodeling is the natural process by which it dynamically maintains its mechanical properties, skeletal integrity and calcium homeostasis. This series of events occurs throughout life and is a well‐regulated process involving bone resorption by osteoclasts and bone formation by osteoblasts. An imbalance in this process can lead to excessive bone loss such as osteoporosis (Katsimbri 2017).

It has been proposed that there is a relationship between incretins and bone remodeling (Mabilleau 2015; Ramsey and Isales 2017). The GIP receptor is widely expressed in bone. Furthermore, bone quality is severely affected in mouse models of GLP‐1R knockout mice and in mice deficient in GIP or its receptor, suggesting there may be a relationship between gut hormones and bone regulation. GIP knockout mice show higher numbers of osteoclasts and thinner trabeculae at 8 weeks of age as well as lower BMD at all time points studied (Xie et al. 2005; Tsukiyama et al. 2006). In an osteoblast cell line, GIP induces cAMP expression in osteoblasts that induces bone formation (Tsukiyama et al. 2006). In humans, a GIP single polymorphism is associated with low mineral bone density at the femoral neck and hip in a Danish perimenopausal women cohort (Torekov et al. 2014). The mechanism by which GIP results in increased bone formation and decreased bone resorption in response to a meal is thought to be due to its ability to promote efficient ingested calcium storage into bone (Xie et al. 2005).

The GLP‐1R is expressed in the pancreas, kidney, lung, heart, stomach, intestine, and CNS (Drucker and Nauck 2006) and may also be expressed in bone. Evidence of GLP‐1R mRNA has been demonstrated in certain osteoblastic cell lines, and GLP‐1R protein has been identified in primary murine osteoblasts (Nuche‐Berenguer et al. 2010; Pacheco‐Pantoja et al. 2011). Furthermore, GLP‐1R knockout mice have been shown to have cortical osteopenia and bone fragility as well as increased osteoclasts and bone resorption (Yamada et al. 2008). When compared to wild‐type animals, GLP‐1‐deficient mice have a significant reduction in bone strength and quality (Mabilleau et al. 2013). Administration of GLP‐1 in diabetic rats results in increased trabecular bone mass and also when administered to nondiabetic osteoporotic ovariectomized OVX rats (Lu et al. 2015; Pereira et al. 2015). In humans, however, incretins do not appear to exert a significant effect in BMD or affect fracture risk. The Liraglutide Effect and Action in Diabetes study was a double‐blind multi‐center randomized control trial that investigated total BMD change from baseline in 746 patients aged 19–79 with T2DM on the sulfonylurea glimepiride, or the GLP‐1 agonist liraglutide at two different doses. The study found no significant differences in BMD among the groups treated with placebo or any dose of liraglutide after 52 or 104 weeks (Gilbert et al. 2016). Two recent meta‐analyses show that treatment with GLP‐1 receptor agonists do not modify the risk for bone fracture compared to other anti‐diabetic drugs or placebo in patients with T2DM (Mabilleau et al. 2014; Driessen et al. 2017). Thus, determination of whether incretin‐based therapies significantly improve fracture risk in patients who already have diabetes warrants continued investigation.

### The effect of obesity and weight loss on bone

**Figure 1:**
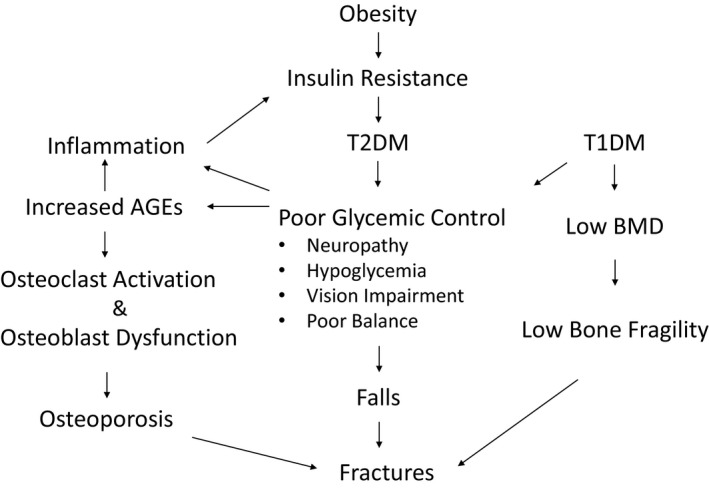
Relationship between obesity, diabetes, inflammation, and bone health. Obesity is associated with insulin resistance and a pro‐inflammatory state that is associated with T2DM. In uncontrolled Type 1 and Type 2 diabetes several factors can worsen bone health; these include increased advanced glycation end products (AGEs) that result in osteoclast activation and osteoblast dysfunction leading to osteoporosis. Neuropathy, vision loss, and poor balance from prolonged poor glycemic control can increase fall risk, this coupled with increased bone fragility associated with Type 1 diabetes can further increase fracture risk.

Total adiposity has positively correlated with BMD (Zhao et al. 2008). This makes sense since heavier bodies require a stronger frame. The mechanisms accounting for this phenomenon are thought to be due to increased loading, the effects of leptin and increased aromatase activity given estrogen's positive effect on bone formation. However, at a certain point, increased adiposity can lead to low BMD and increase the risk for vertebral fractures (Kim et al. 2010; Compston et al. 2011). Furthermore, T1DM or T2DM, independently increase fracture risk (Napoli et al. 2017). The reasons for this appear to be multi‐factorial and involve impaired glucose control with subsequent complications leading to osteocyte dysfunction, increased bone fragility, and increased inflammation mediated by cytokine production and advanced glycation end products (AGEs). In T1DM, individuals have been found to have decreased BMD that is unrelated to HgA1c level (Eller‐Vainicher et al. 2011). In both T1DM and T2DM, complications stemming from poor glycemic control such as neuropathy, poor vision, balance impairment, and hypoglycemia can increase fall risk resulting in a higher fracture risk (Napoli et al. 2017). Inflammatory cytokines such as TNF‐*α* and IL‐6 which are associated with diabetes and obesity have been shown to cause osteoblast dysfunction. Furthermore, these cytokines are associated with central or visceral fat deposition as opposed to a peripheral fat metabolic profile, a profile with a deleterious effect on BMD (Cohen et al. 2013; Walsh and Vilaca 2017). Furthermore, the accumulation of AGEs negatively affects bone remodeling through the formation of cross‐linked protein aggregates and by directly activating inflammation and oxidative stress pathways that promote osteoporosis (Fig. 1) (Sanguineti et al. 2014).

### Bone health after bariatric surgery

Fat soluble micronutrient absorption is significantly affected by bariatric surgery given its effect on fat absorption and dietary intake restriction. In both the SG and RYGB‐reduced dietary intake leads to less consumption of foods containing calcium and vitamin D. RYGB surgery may predispose to more vitamin D malabsorption than SG due to the nature of intestinal rearrangement. Bile and pancreatic enzymes do not reach ingested food and supplements until the Roux limb and biliopancreatic limbs join to form the common channel (Schafer 2017). A retrospective cohort study that analyzed over 1000 patients at 1 and 5 years after bariatric surgery found that the prevalence of secondary hyperparathyroidism, a readout of impaired calcium and vitamin D absorption, was 56.6% in patients who had undergone RYGB and 41.7% in patients who had undergone an SG 5 years out of surgery. Interestingly, 21% of these patients already had secondary hyperparathyroidism before surgery, which was associated with presurgical vitamin D deficiency (Wei et al. 2018). Vitamin D deficiency is common in obese patients with reported incidences in the 50 sec–96% (Vix et al. 2014). Reasons accounting for vitamin D deficiency in the setting of obesity include increased vitamin D absorption by adipose tissue, reduced liver synthesis of vitamin D due to hepatosteatosis, reduced exposure to sunlight, reduced cutaneous synthesis in dark‐skinned individuals, and an increase in vitamin D clearance in the setting of chronic inflammation (Yanoff et al. 2006; Compher et al. 2008; Schafer 2017).

Weight loss results in a decrease in estrogen, leptin, and insulin, and an increase in adiponectin, which can negatively impact bone health. Nonsurgical weight loss has been associated with a reduction of total body BMD after 6 months (Zibellini et al. 2015), and a meta‐analysis of 32 randomized controlled trials found a significant reduction of hip and lumbar spine BMD after diet‐induced weight loss after 4 months (Soltani et al. 2016). Therefore, weight loss in general is associated with a decrease in BMD likely due to the changes in adipokines and endocrine hormones that occur after weight loss. Whether bariatric surgery‐induced weight loss results in a distinct alteration in bone metabolism will depend on the changes in the hormonal milieu that occur specifically in the event of bariatric surgery (Table 1) (Ma et al. 2016; Duggan et al. 2019; Wolf et al. 2019). These include the rise of incretins such as GLP‐1 and PYY and any nutritional deficiencies that are specific to postbariatric patients. While studies in mice suggest that GIP and GLP‐1 may exert a positive effect on bone, elevations of PYY have been associated with decreased bone turnover in adolescent girls with anorexia nervosa (Misra et al. 2006) Several studies have shown that there is an association between bariatric surgery and a decrease in bone mass (von Mach et al. 2004). Bariatric surgery can lead to significant metabolic and nutritional deficiencies which can negatively impact bone metabolism (Folli et al. 2012). Calcium and vitamin D malabsorption has been a major concern after bariatric surgery since the primary site for calcium absorption is the duodenum and proximal jejunum, and the jejunum, and ileum are the site for vitamin D absorption. Reduction in the small intestinal surface area can eventually result in secondary hyperparathyroidism and osteomalacia (Goode et al. 2004; Al‐Shoha et al. 2009). Evidence from epidemiological studies suggest that malabsorption from certain restrictive and malabsorptive bariatric surgery procedures such as the RYGB and biliopancreatic diversion with DS are associated with increased risk of fractures that may begin to manifest between 2 and 5 years after surgery (Rousseau et al. 2016; Gagnon and Schafer 2018). There are insufficient studies to conclude whether fracture risk increases with the sleeve gastrectomy (Gagnon and Schafer 2018). However, bone turnover markers do rise after bariatric surgery (von Mach et al. 2004). CTX, a biochemical marker of bone resorption, increases as early as 10 days postoperatively and peaks by 6‐12 months after RYGB (Yu et al. 2016), and postbariatric surgery bone turnover markers rise significantly more in patients who undergo RGYB compared to SG (Ivaska et al. 2017). Evidence of bone loss after bariatric surgery has been demonstrated by dual‐energy x‐ray absorptiometry (DXA) in a multitude of studies (von Mach et al. 2004; Nogues et al. 2010; Casagrande et al. 2012; Stein et al. 2013; Stein and Silverberg 2014; Yu et al. 2014, 2016). However, it is worth noting that DXA is not highly accurate in the setting of obesity or rapid weight loss (Yu et al. 2014) Nonetheless, studies with high‐resolution peripheral quantitative CT which may be less affected by changes in fat, have demonstrated volumetric BMD declines particularly at the tibia and in cortical bone 1 year after RYGB (Stein et al. 2013). However, many of the studies looking at fracture risk after bariatric surgery have been done in women. Thus, the reduction in bone mass observed may be largely influenced by the decline in estrogen levels after surgery. Indeed, a recent study showed increased cortical porosity at the radius and tibia 12 months after RYGB and these skeletal detriments were worse in postmenopausal women (Schafer et al. 2018).

## Reproductive Hormones and Fertility

**Figure 2:**
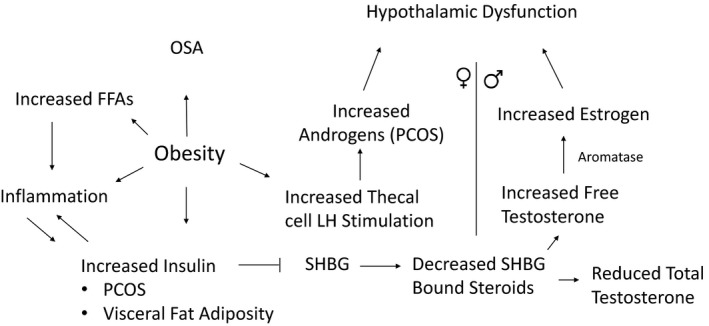
The association between obesity, insulin resistance, and reproduction. Increased adiposity, in particular central obesity is associated with increased insulin resistance which leads to a reduction in sex hormone‐binding globulin (SHBG) production. This leads to a reduction in SHBG‐bound steroids such as total testosterone thereby resulting in an increase in free testosterone. In women, androgens are further increased by increased theca cell sensitivity which occurs in the setting of obesity and insulin resistance, a feature associated with PCOS. Androgen excess is a major cause of ovarian dysfunction and ovulatory disturbance that eventually leads to hypothalamic dysfunction in PCOS. In obese men, increased levels of free testosterone are converted to estrogen by aromatase in the setting of an abundance of adipose tissue, thus promoting inhibition of LH secretion by the pituitary which results in hypothalamic dysfunction. In both men and women, insulin resistance is associated with obstructive sleep apnea and increased inflammation which aggravate insulin resistance and perpetuate a hypogonadal state.

The impact of obesity on reproductive function in both women and men has been extensively documented (Pasquali and Gambineri 2006). The association between obesity and anovulation has been known for over 40 years (Hartz et al. 1979). Furthermore, ovulatory infertility has also been reported with increasing BMI (Rich‐Edwards et al. 1994). Obesity is associated with subfecundity and infertility related to abnormalities in reproductive hormones, egg quality as well as endometrial receptivity (Talmor and Dunphy 2015). Obesity can negatively affect the outcome of assisted reproductive technologies (Provost et al. 2016). In women, obesity is well known to be associated with higher rates of polycystic ovary syndrome (PCOS) (Rosenfield and Ehrmann 2016), while high levels of insulin have been implicated in the pathogenesis of PCOS (Pasquali 2006; Rosenfield and Ehrmann 2016; Durmus et al. 2017; Glueck and Goldenberg 2019). In PCOS, insulin resistance is present independent of obesity but is exacerbated by the presence of obesity (Dunaif et al. 1989, 1992; Glueck and Goldenberg 2019). Increased visceral adiposity has been demonstrated in women with PCOS associated with higher levels of inflammatory markers (Pasquali 2006; Durmus et al. 2017). Even nonobese women with PCOS have been shown to have an increase in abdominal fat depots compared to control women of the same BMI (Huang et al. 2013). Furthermore, adipose tissue from women with PCOS exhibit an enhanced lipolytic catecholamine response (Ek et al. 2002). Enhanced visceral fat lipolysis promotes increased free fatty acid release into the portal circulation and increased hepatic insulin resistance (Samuel et al. 2010). Insulin resistance activates inflammatory pathways such as nuclear factor kB (NF‐κB) and c‐Jun N‐terminal kinase (JNK) which can also cause insulin resistance and in this way perpetuate a vicious cycle of inflammation and insulin resistance (Fig. 2) (Yuan et al. 2001; Hirosumi et al. 2002). Furthermore, sex hormone secretion and metabolism are largely influenced by obesity and fat distribution. Higher circulating insulin levels in centrally obese women result in reduced SHBG synthesis in the liver which causes in a higher percentage of free testosterone in these women. A reduction in SHBG‐bound androgens, results in a compensatory increase in the metabolism of androgens in female obesity (Samojlik et al. 1984; Kurtz et al. 1987). In PCOS, ovarian function is hypersensitive to LH stimulation, thereby magnifying ovarian hyperandrogenism (Rosenfield and Ehrmann 2016; Glueck and Goldenberg 2019). Hypothalamic dysfunction subsequently occurs in PCOS due to increased pulse frequency of GnRH related to higher circulating androgen levels that reduce sensitivity to progesterone feedback leading to preference for secretion of LH compared to FSH (McCartney and Marshall 2016).

In men, obesity is associated with low testosterone together with low sex hormone‐binding globulin (SHGB) and increased estradiol. This is usually accompanied by low or inappropriately normal gonadotropins, a conditioned termed male obesity secondary hypogonadism (MOSH), which occurs in about 40% of obese men (Saboor Aftab et al. 2013). A reduction in SHBG is a major reason for the reduction in free testosterone in obese men. Furthermore, aromatization of free testosterone to estrogen occurs in the presence of increased adipose by aromatase which leads to inhibition of LH secretion by the pituitary (Hofstra et al. 2008). Leptin has also been shown to inhibit LH thereby contributing to hypothalamic hypogonadism in obese men (Fig. 2) (Isidori et al. 1999).

## Metabolic Implication of Weight Loss for PCOS, Fertility, and Bone Health

Weight loss is the most effective strategy for the treatment of PCOS and subsequent improvement of insulin sensitivity. A recent meta‐analysis involving 13 studies of 2130 women with PCOS has examined the impact of bariatric surgery and suggests that the reproductive features of PCOS are significantly improved after 12 months (Skubleny et al. 2016). Nonalcoholic fatty liver disease (NAFLD) is a rising comorbidity associated with patients that are obese or have metabolic syndrome components such as T2DM or hyperlipidemia. Interestingly, bariatric surgery has been shown to lead to improvement in nonalcoholic steatohepatitis (NASH), inflammation and fibrosis in patients with biopsy proven NASH (Lassailly et al. 2015; Shouhed et al. 2017).

Obesity is associated with reduced libido in both men and women as well as erectile dysfunction (ED) (Kolotkin et al. 2012). A recent meta‐analysis found that gonadal dysfunction was quite prevalent in obese women and men who presented for bariatric surgery (Escobar‐Morreale et al. 2017). In the female patients with PCOS (36% of women), 96% of them experienced resolution after bariatric surgery. However, of the 64% of affected males with MOSH, 87% experienced resolution of gonadal dysfunction (Escobar‐Morreale et al. 2017). A prospective weight loss study looking at 97 men who underwent RYGB found that on average self‐reported sexual function measures in all categories, including ED and sexual drive, significantly improved after bariatric surgery. The authors found the amount of weight loss independently predicted the degree of improvement in all domains (Dallal et al. 2008). In a prospective study done in 54 women who had undergone the laparoscopic adjustable gastric band restrictive procedure or RYGB, the authors found that 68% of women had self‐reported scores indicating resolution of female sexual dysfunction 6 months postbariatric surgery. Participants’ Female Sexual Function Index Scores were indistinguishable from normative controls after surgery (Bond et al. 2011). While weight loss improves fertility and is associated with lower rates of gestational diabetes, there is some evidence for an association with having short for gestational age births in pregnant women who are postbariatric surgery when compared to age matched pregnant controls (Johansson et al. 2015). Therefore, women who have undergone bariatric surgery are urged not to become pregnant 1–1.5 years after bariatric surgery and their nutritional status should be optimized prior and during pregnancy (Falcone et al. 2018).

There is a close relationship between metabolic parameters, the gonadal axis and bone health. Gut hormones allow for anabolic processes such as the optimal storage and utilization of nutrients by bone cells. These processes are affected by the amount of adiposity which positively correlates to the levels of insulin. Insulin resistance is associated with inflammation, gonadal dysfunction, and eventually a deleterious effect on bone health.

**Figure 3:**
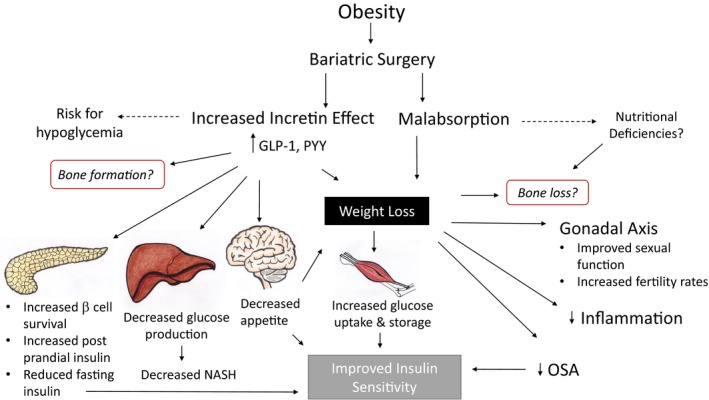
Combined effects after bariatric surgery on incretins, metabolic parameters, and bone health. Bariatric surgery is the most effective treatment for sustained weight loss in morbid obesity. The anatomical rearrangements lead to an increase in incretins which ultimately result in improved metabolic parameters associated with weight loss and improved insulin sensitivity.

However, bowel rearrangement by bariatric surgery allows for the rise of incretins such as GLP‐1 and PYY that contribute to sustained weight loss due to their incretin effect as well as their satiety signals to the brain. This results in improved insulin sensitivity and may increase bone formation. However, because weight loss and leptin reduction are both associated with bone loss, the eventual net outcome on bone health after bariatric surgery will depend on many factors which will likely include the amount of weight loss, the presence of any nutritional deficiencies, and the level of gonadal hormones after bariatric surgery. Nevertheless, the implications of weight loss on organs such as muscle, pancreas and liver will lead to marked improvement in peripheral insulin sensitivity, which usually results in the remission of T2DM as well as improvement in gonadal function (Fig. 3).

## Conclusions

Bariatric surgery is the most effective treatment for sustained weight loss in patients with morbid obesity. Surgical rearrangement and restriction lead to a rise in incretin hormones that result in increased satiety, improved insulin sensitivity, and profound weight loss which leads to the reduction of T2DM, obstructive sleep apnea, hepatic steatosis, inflammation and improved fertility including in patients with PCOS. However, bariatric surgery may result in bone loss due to potential vitamin D deficiency, mechanical unloading from weight loss, and a change in hormonal secretion such as a reduction in leptin, and estrogen that may be more pronounced in women who may be perimenopausal. Further studies looking at long‐term outcomes on bone health after bariatric surgery are needed to understand these potential effects.

## Conflict of Interest

The authors have nothing to disclose.
